# Simulated Nitrogen Deposition Reduces CH_4_ Uptake and Increases N_2_O Emission from a Subtropical Plantation Forest Soil in Southern China

**DOI:** 10.1371/journal.pone.0093571

**Published:** 2014-04-08

**Authors:** Yongsheng Wang, Shulan Cheng, Huajun Fang, Guirui Yu, Minjie Xu, Xusheng Dang, Linsen Li, Lei Wang

**Affiliations:** 1 Key Laboratory of Ecosystem Network Observation and Modeling, Institute of Geographical Sciences and Natural Resources Research, Chinese Academy of Sciences, Beijing, China; 2 University of Chinese Academy of Sciences, Beijing, China; Tennessee State University, United States of America

## Abstract

To date, few studies are conducted to quantify the effects of reduced ammonium (NH_4_^+^) and oxidized nitrate (NO_3_^−^) on soil CH_4_ uptake and N_2_O emission in the subtropical forests. In this study, NH_4_Cl and NaNO_3_ fertilizers were applied at three rates: 0, 40 and 120 kg N ha^−1^ yr^−1^. Soil CH_4_ and N_2_O fluxes were determined twice a week using the static chamber technique and gas chromatography. Soil temperature and moisture were simultaneously measured. Soil dissolved N concentration in 0–20 cm depth was measured weekly to examine the regulation to soil CH_4_ and N_2_O fluxes. Our results showed that one year of N addition did not affect soil temperature, soil moisture, soil total dissolved N (TDN) and NH_4_^+^-N concentrations, but high levels of applied NH_4_Cl and NaNO_3_ fertilizers significantly increased soil NO_3_^−^-N concentration by 124% and 157%, respectively. Nitrogen addition tended to inhibit soil CH_4_ uptake, but significantly promoted soil N_2_O emission by 403% to 762%. Furthermore, NH_4_^+^-N fertilizer application had a stronger inhibition to soil CH_4_ uptake and a stronger promotion to soil N_2_O emission than NO_3_^−^-N application. Also, both soil CH_4_ and N_2_O fluxes were driven by soil temperature and moisture, but soil inorganic N availability was a key integrator of soil CH_4_ uptake and N_2_O emission. These results suggest that the subtropical plantation soil sensitively responses to atmospheric N deposition, and inorganic N rather than organic N is the regulator to soil CH_4_ uptake and N_2_O emission.

## Introduction

Humid tropical biome stores approximately 10% of global soil carbon (C) [1] and plays a vital role in the budget of ecosystem C and nitrogen (N) fluxes. The amount of nitrous oxide (N_2_O) emission from the subtropical and tropical forest soils is estimated at 0.9–3.6 T g yr^−1^, accounting for 14% to 23% of the global N_2_O budget [2]. Simultaneously, well-aerated soils in the subtropical and tropical forests potentially function as a significant sink of atmospheric methane (CH_4_) during the dry season [3]–[6]. The uptake of CH_4_ from the subtropical and tropical forest soils is estimated to be 6.2 T g yr^−1^, accounting for 28% of the global CH_4_ sink [7]. Although the importance of the subtropical and tropical forest soils as atmospheric CH_4_ sink and N_2_O source is well understood, few observations can be available in this region [8]–[10]. Moreover, low-frequency measurement of gas fluxes in the few studies is unable to accurately estimate the annual amount of soil CH_4_ uptake and N_2_O emission, which leads to a high uncertainty in the budget of global soil CH_4_ and N_2_O fluxes.

Chronic N deposition input into terrestrial ecosystems alters plant physiology and soil microbial community, thereby changes the soil biogenic CH_4_ and N_2_O fluxes [11]–[13]. Based on a meta-analysis of N addition experimental data in globe, Liu and Greaver [14] concluded that N addition reduced CH_4_ uptake by 38% and increased N_2_O emission by 216%. In general, chronic N deposition will increase NH_4_^+^and NO_3_^−^ availability in the forest ecosystems, thereby affects CH_4_ uptake from the forest soils through changing the activity and composition of methanotrophic community [15]–[17]. Soil NH_4_^+^ accumulation can decrease, increase or have no effects on soil CH_4_ uptake in the forest ecosystems, depending on forest types [5], duration of N application [18], and N fertilizer types and doses [19]. Three potential mechanisms have been proposed to clarify the inhibition of NH_4_^+^ accumulation to soil CH_4_ uptake: (1) the competition of soil NH_3_ to use CH_4_ monooxygense with soil CH_4_ [20], (2) the toxic inhibition of hydroxylamine and nitrite produced during soil NH_4_^+^ oxidation [21], and (3) the indirect effects of applied N and associated salt ions through osmotic stress [22]. On the contrary, elevated soil NH_4_^+^ availability can increase soil CH_4_ uptake, which is related to the increase in the quantity of soil ammonia-oxidizing microorganisms [23]. Soil NO_3_^−^ accumulation can also decrease or increase soil CH_4_ uptake [18], [24]. Osmotic stress caused by NO_3_^−^-N fertilizer-associated salts [22] and anaerobic ‘microsites’ produced by NO_3_^−^ reduction [25] are toxic to CH_4_-oxidizing bacteria. The mechanism responsible for the promotion of NO_3_^−^ addition to soil CH_4_ uptake is still unclear and need a number of experimental evidences to support [26]. A positive relationship between the amount of N addition and N_2_O fluxes from the subtropical forest soils is mainly attributed to the promotion of soil nitrification or/and denitrification rates caused by increased N availability [27], [28]. Some studies reported that denitrification was the main source of N_2_O emission from the subtropical forest soils [29]–[31], whereas other studies claimed that nitrification dominated soil N_2_O fluxes [32]. To date, single N fertilizer (i.e., NH_4_NO_3_) is widely used to simulate the effects of N deposition in all N manipulative experiments of subtropical forests in China [8], [33], [34]. The above studies have not evaluated the relative contributions of the deposited N ions (NH_4_^+^ and NO_3_^−^) to soil CH_4_ uptake and N_2_O emission. Moreover, most of soil CH_4_ and N_2_O fluxes are measured by low-frequency sampling over the short term, which is difficult to accurately assess the budget of soil CH_4_ and N_2_O fluxes and leads to great uncertainty.

In China, the plantations cover an area of 6.2×10^7^ ha, accounting for 31.8% of China's forest area and ranking first in the world [35]. Approximately 63% of plantations concentrate in the subtropical region of southern China [36]. Meanwhile, the southern China is the most economically developed regions with high population density, and plantations, cities and farmlands are interspersed. Because a number of reactive N originated from fossil fuel combustion and fertilizer use is emitted to atmosphere, the forests in this region are receiving a high level of anthropogenic N deposition, mostly as ammonium [37]. Atmospheric N deposition rate via precipitation in southern China has been reported and ranges from 30 to 73 kg N ha^−1^ yr^−1^ [8]. So far, few studies are conducted to examine the effects of N deposition on CH_4_ uptake and N_2_O emission from the plantation of this region [33], [34].

Humid subtropical forest soils are generally characterized by high N availability and high N turnover [38]. Therefore, we hypothesize that increased NH_4_^+^ and NO_3_^−^ availability via experimental N deposition will inhibit soil CH_4_ uptake and promote N_2_O emissions from the subtropical plantation. Furthermore, NH_4_^+^-N fertilizer addition will decrease CH_4_ uptake and increase N_2_O emission due to soil NH_4_^+^-N accumulation. In contrast, the effects of NO_3_^−^-N fertilizer addition on soil CH_4_ uptake and N_2_O emission depend on the concentration of soil NO_3_^−^-N as well as associated salt ions. Our objectives were (1) to quantify the effects of NH_4_^+^-N and NO_3_^−^-N fertilizer application on soil CH_4_ and N_2_O fluxes and soil variables in the subtropical plantation; (2) to examine the relationships between soil CH_4_ and N_2_O fluxes and the relevant soil properties.

## Materials and Methods

### Site description

This study was conducted in a subtropical slash pine plantation at the Qianyanzhou Ecological Station (QYZ, 26°44′39″N, 115°03′33″E) in southern China. The station belongs to the Institute of Geographic Sciences and Natural Resources Research, Chinese Academy of Sciences. All necessary permits were obtained for this field study. The field study did not involve endangered or protected species. According to local climate records from 1989 to 2008, mean temperature of QYZ site varies between 17 and 19°C. Mean annual precipitation ranges from 945 to 2145 mm, of which 24%, 41%, 23% and 12% occurs in four quarters in turn. The rainfall scarcity and high temperature in late summer often result in seasonal drought [39]. The exotic slash pine plantation was established in 1985. Mean tree height, diameter at breast height, stand basal area, and leaf area index were 12.0 m, 15.8 cm, 35 m^2^ ha^−1^, and 4.5 m^2^ m^−2^, respectively [40]. The main understory and midstory species are *Woodwardia japonica* (L.f.) Sm., *Dicranopteris dichotoma* (Thunb) Bernh, *Loropetalum chinense* (R.Br.) Oliv, and *Quercus fabric* Hance. The red soil is weathered from red sand rock, and soil texture is divided into 2.0-0.05 mm (17%), 0.05-0.002 mm (68%), and <0.002 mm (15%) [39].

### Experimental design

The N addition experiment is a randomized block design. In May 1, 2012, two N fertilizers (NH_4_Cl and NaNO_3_) were used to simulate the effects of deposited NH_4_^+^ and NO_3_^−^ on ecosystem processes and functions. According to the level of atmospheric N deposition at the QYZ site, two levels referred to as low N (40 kg N ha^−1^ yr^−1^) and high N (120 kg N ha^−1^ yr^−1^) were used to simulate a future increase in the atmospheric N deposition by 1-, and 3-fold. A control treatment was designed at each block to calculate the net effect of N addition. Each N treatment was replicated three times, and a total of 15 plots were included. Each plot with 20 m×20 m was divided into four subplots with 5 m×5 m, and the plots were separated by 10 m wide buffer strips. Three subplots were used to measure soil CH_4_ and N_2_O fluxes, and the other one was used to investigate aboveground biomass and diversity. N fertilizer solutions were sprayed on the plots once a month in 12 equal applications over the entire year, and the control plots received equivalent deionized water only.

### Measurement of soil CH_4_ and N_2_O fluxes

Flux measurements of soil CH_4_ and N_2_O fluxes were performed by using a static opaque chamber and gas chromatography method [41]. The static chambers were made of stainless steel and consisted of two parts: a square base frame (length×width×height = 50 cm×50 cm×10 cm) and a removable top (length×width×height = 50 cm×50 cm×15 cm). The installed equipments on the static chambers were detailed by Fang et al. [42]. The frames were inserted directly into the soil to a depth of 10 cm and remained intact during the entire observation period. To assess the spatial heterogeneity of soil C and N fluxes, a pre-experiment was conducted to examine the difference of CH_4_ and N_2_O fluxes among the three subplots of each plot before N addition. No significant difference of CH_4_ and N_2_O fluxes among the three subplots was found during the observation, suggesting a negligible effect of soil heterogeneity. Considering the practical reasons such as high labor intensity, we collected gas samples through changing the subplots within a month. The soil CH_4_ and N_2_O fluxes were measured twice a week and conducted between 9:00 and 11:00 am (China Standard Time, CST). Five gas samples were taken using 100 ml plastic syringes at intervals of 0, 10, 20, 30, and 40 min after closing the chambers. CH_4_ and N_2_O concentrations of gas samples were analyzed within 24 h with a gas chromatography (Agilent 7890A, USA) equipped with an electron capture detector (ECD) for N_2_O analysis and a flame ionization detector (FID) for CH_4_ analysis. The high purity N_2_ and H_2_ were used as carrier gas and fuel gas, respectively. The ECD and FID were heated to 350°C and 200°C, respectively, and the column oven was kept at 55°C. The soil fluxes were calculated based on their rate of concentration change within the chamber, which was estimated as the slope of linear or nonlinear regression between concentration and time [41]. All the coefficients of determination (r^2^) of the regression were greater than 0.90 in our study.

### Measurements of soil temperature and moisture

Simultaneously, soil temperature at 5 cm (T_s_) and soil moisture at 10 cm below soil surface (SM) were monitored at each chamber. Soil temperature was measured using a portable temperature probes (JM624 digital thermometer, Living–Jinming Ltd., China). Volumetric soil moisture (m^3^ m^−3^) was measured using a moisture probe meter (TDR100, Spectrum, USA).

### Soil sampling and mineral N analysis

During the measurement of soil CH_4_ and N_2_O fluxes, soil samples were collected weekly nearby the static chambers from a depth of 0–20 cm using an auger (2.5 cm in diameter). Five soils were collected and were pooled to one composite sample for each soil layer at each plot. Soils were immediately passed through a 2 mm sieve to remove roots, gravel and stones. Soil samples were extracted in 1.0 M KCl solution (soil: water = 1∶10) and shaken for 1 h. The soil suspension was subsequently filtered through Whatman No. 40 filter papers for NH_4_^+^-N, NO_3_^−^-N, and total dissolved nitrogen (TDN) determination on a continuous-flow autoanalyzer (Seal AA3, Germany). Dissolved organic nitrogen (DON) concentration was calculated as the difference between TDN and total inorganic nitrogen (NH_4_^+^-N and NO_3_^−^-N).

### Statistical analyses

Repeated measures analysis of variance (AVOVA) with Duncan test was applied to examine the differences of soil temperature, soil moisture, soil dissolved N, and soil CH_4_ and N_2_O fluxes between control and N addition plots. Experimental treatments were set as factors of between-subjects and measurement date was selected as a variable of within-subjects. Linear and nonlinear regression analyses were used to examine the relationships between soil CH_4_ and N_2_O fluxes and the measured soil variables in monthly scale. All statistical analyses were conducted using the SPSS software package (version 16.0), and statistical significant differences were set with *P* values<0.05 unless otherwise stated. All figures were drawn using the Sigmaplot software package (version 10.0).

## Results

### Soil temperature, moisture and precipitation

**Figure 1:**
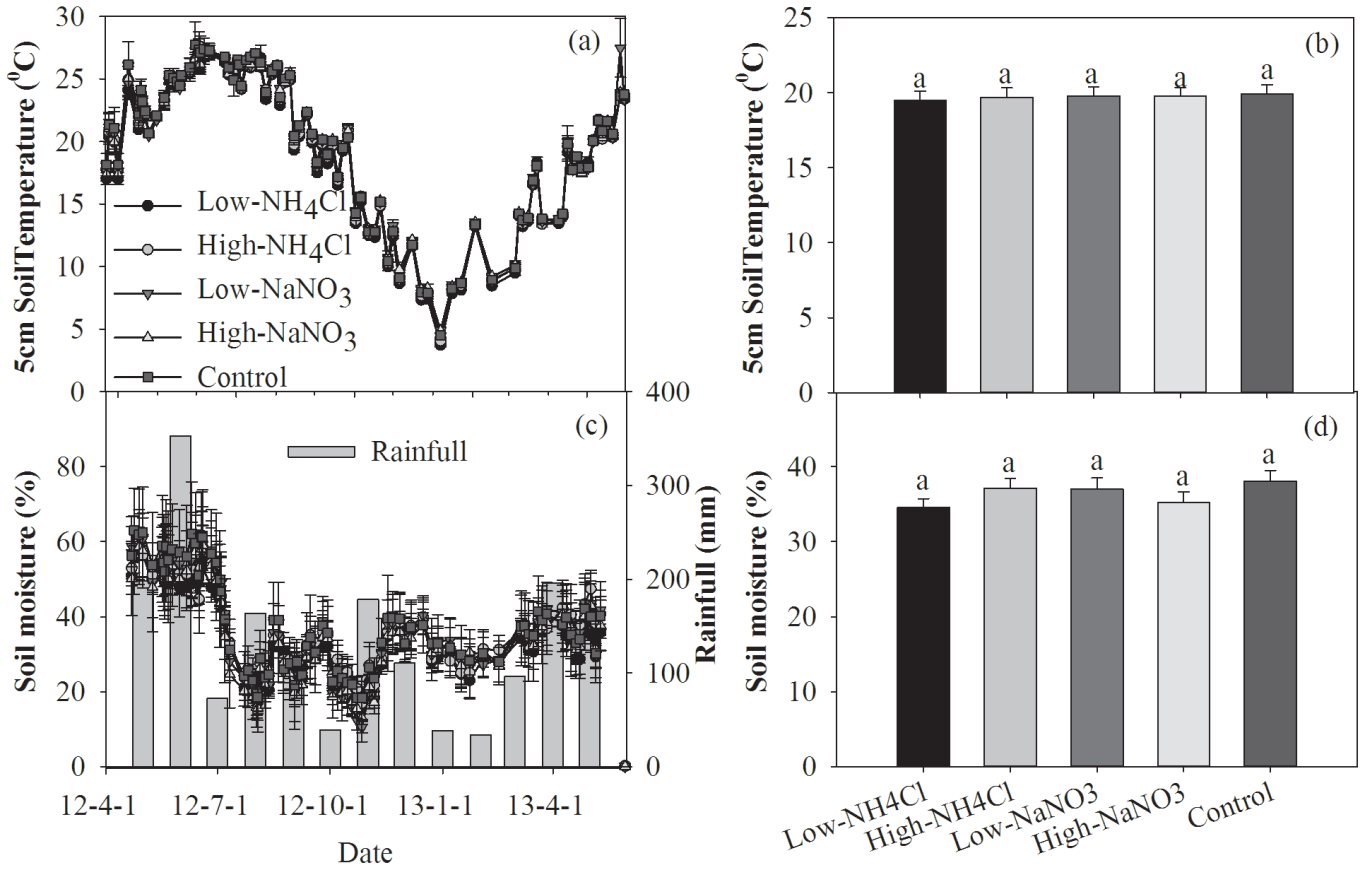
Temporal variations of 5 cm soil temperature and 10 cm soil moisture and their responses to N addition. Different letters above the columns mean significant differences between experimental treatments.

During the whole observation period, soil temperature at 5 cm depth fluctuated greatly, which correlated with the weather condition. Soil temperature varied as a single-peak and single-sink curve, i.e. temperature was the highest in early July, gradually reached the lowest value in early January, and then increased (Fig. 1a). There was no significant difference in surface temperature among various treatments (Fig. 1b).

Soil moisture at 10 cm depth behaved as significant seasonal variation, with the maximum occurred in May and June and the minimum occurred in August and October (Fig. 1c). The seasonality of soil moisture was well consistent with that of precipitation (Fig. 1c). Similar to surface temperature, no significant difference in soil moisture was found among various treatments (Fig. 1d).

### Soil dissolved N concentrations

**Figure 2:**
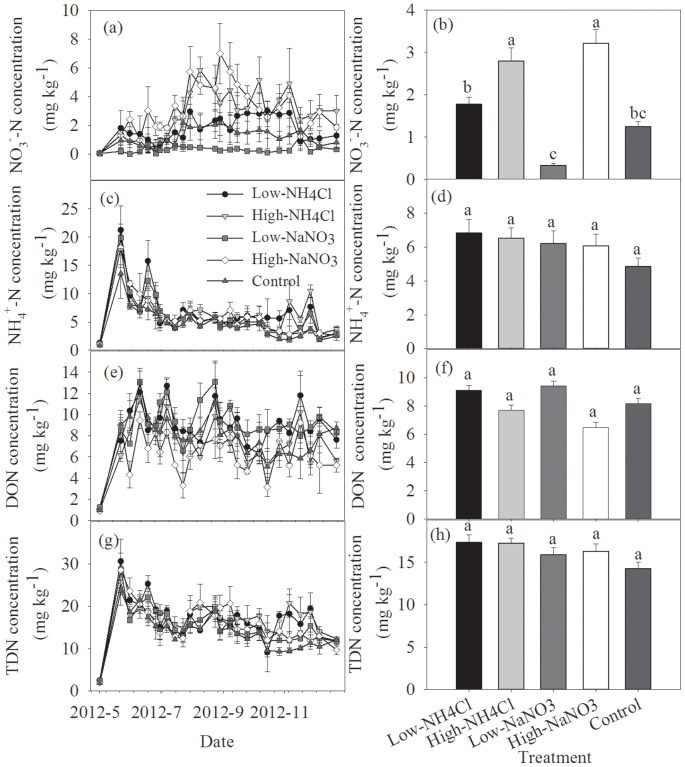
Temporal variations of soil NO_3_^−^-N, NH_4_^+^-N, DON, and TDN concentrations and their responses to N addition. Different letters above the columns mean significant differences between experimental treatments.

Soil NO_3_^−^-N concentration showed significant seasonal variation, with the minimum and maximum occurring in May and August (Fig. 2a, Table 1, *P* = 0.016). In the control, the concentration of soil NO_3_^−^-N ranged from 0.06 to 2.19 mg kg^−1^, with an average of 1.25 mg kg^−1^ (Fig. 2a–b). N addition tended to alter soil NO_3_^−^-N concentration, and the difference was significant among five experimental treatments (Table 1, *P* = 0.026). Compared with the control, high level of NaNO_3_ addition tended to increase soil NO_3_^−^-N concentration, while an opposite pattern was found in the low level of NaNO_3_ addition treatment (Fig. 2b). Furthermore, the promotion of high level of NH_4_Cl addition to soil NO_3_^−^-N concentration seemed to be stronger than that of low level of NH_4_Cl addition (Fig. 2b).

**Table 1:** Results of repeated-measures ANOVAs on the effects of experimental treatment, month and their interaction on soil dissolved N concentrations.

Source of variation	Soil NO_3_^−^-N		Soil NH_4_^+^-N		Soil DON		Soil TDN	
	F	p	F	p	F	p	F	p
Month	11.49	0.016	38.30	<0.001	16.71	<0.001	37.97	0.002
Treatment	4.40	0.026	1.62	0.24	1.81	0.20	1.51	0.27
Month×Treatment	1.31	0.24	1.41	0.13	1.33	0.17	0.75	0.77

Soil NH_4_^+^-N concentration peaked in the middle of May, and then continued to decrease (Fig. 2c). The seasonal variation of soil NH_4_^+^-N concentration was significant (Table 1, *P*<0.001). In the control, soil NH_4_^+^-N concentration ranged from 1.91 to 10.80 mg kg^−1^, with an average of 4.84 mg kg^−1^ (Fig. 2d). Overall, although N addition treatments tended to increase soil NH_4_^+^-N concentration, the difference between N addition treatments and control was not significant (Fig. 2d, Table 1, *P* = 0.244).

Soil DON concentration exhibited a significant seasonal variation (Fig. 2e, Table 1, *P*<0.001), and its seasonality was the same as that of soil NO_3_^−^-N concentration (Fig. 2a and Fig. 2e). In the control, soil DON concentration ranged from 5.30 to 14.11 mg kg^−1^, with an average of 8.18 mg kg^−1^ (Fig. 2e–f). Low level of N addition tended to increase the concentration of soil DON, while high level of N addition tended to reduce the concentration of soil DON (Fig. 2f). However, N addition did not change soil DON concentration at the level of 0.05 (Table 1, *P* = 0.203).

The seasonal variation of soil TDN concentration was consistent with that of soil NH_4_^+^-N concentration, dramatically decreasing from May to December (Fig. 2c and Fig. 2g). The seasonal variation of soil TDN concentration was significant (Table 1, *P* = 0.002). N addition tended to increase soil TDN concentration; moreover, the promotion of NH_4_Cl application to soil TDN concentration was slightly higher than that of NaNO_3_ addition (Fig. 2h). However, the difference of soil TDN concentration among the five experimental treatments was not significant (Table 1, *P* = 0.273).

### Soil CH_4_ and N_2_O fluxes

**Figure 3:**
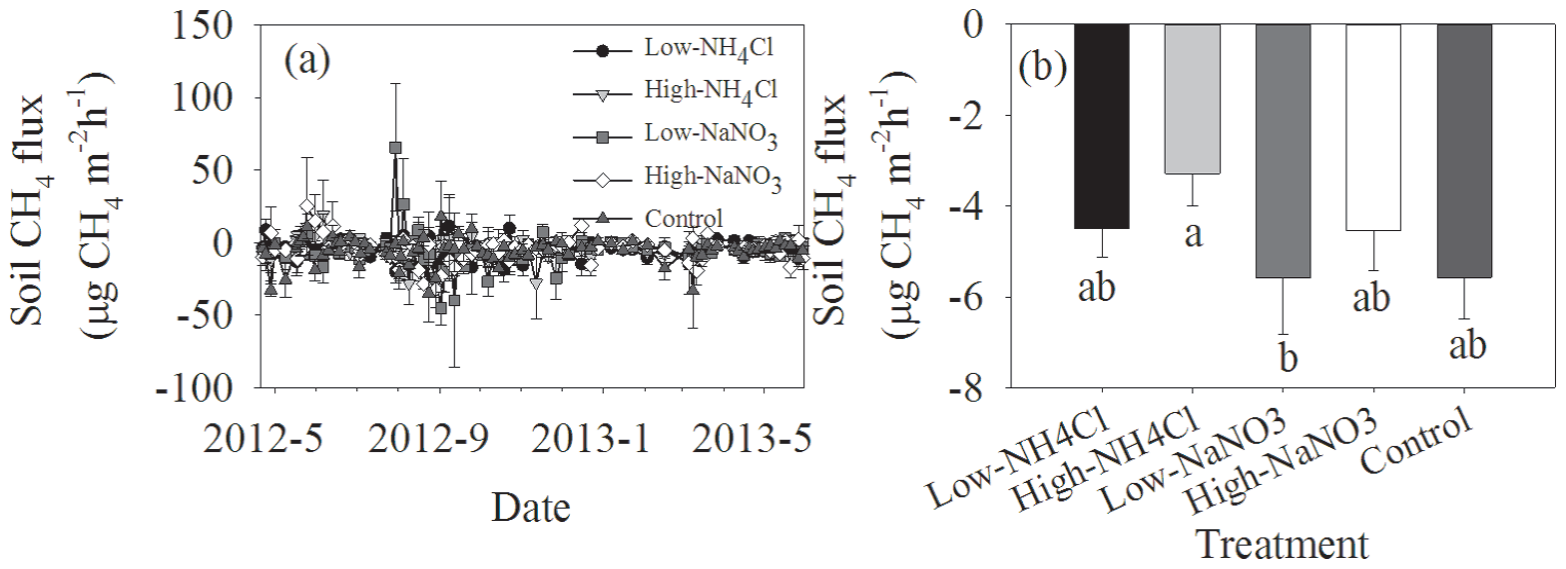
Temporal variations of soil CH_4_ fluxes and their responses to N addition. Different letters below the columns mean significant differences between experimental treatments.

Soil CH_4_ fluxes showed a significant seasonal pattern (Table 2, *P* = 0.008). We observed both soil CH_4_ uptake and emission in the control plots, ranging from −34.9 to 17.9 µg CH_4_ m^−2 ^h^−1^, with an average of −5.56 µg CH_4_ m^−2 ^h^−1^ (Fig. 3a–b). A weak interaction between measurement date and treatment was found (Table 2, *P* = 0.079). Significant differences in CH_4_ fluxes between the control and N addition treatments were only found in July and September (Fig. 3a). For the same level of N addition, NH_4_Cl fertilizer exhibited a higher inhibition to soil CH_4_ uptake than NaNO_3_ fertilizer. However, there was no significant difference in soil CH_4_ fluxes between the control and N addition treatments (Fig. 3b).

**Table 2:** Results of repeated-measures ANOVAs on effects of experimental treatment, month and their interaction on soil CH_4_ and N_2_O fluxes.

Source of variation	CH_4_ flux		N_2_O flux	
	F	p	F	p
Month	2.34	0.008	23.83	<0.001
Treatment	0.66	0.064	2.47	0.011
Month×Treatment	1.37	0.079	2.35	<0.001

**Figure 4:**
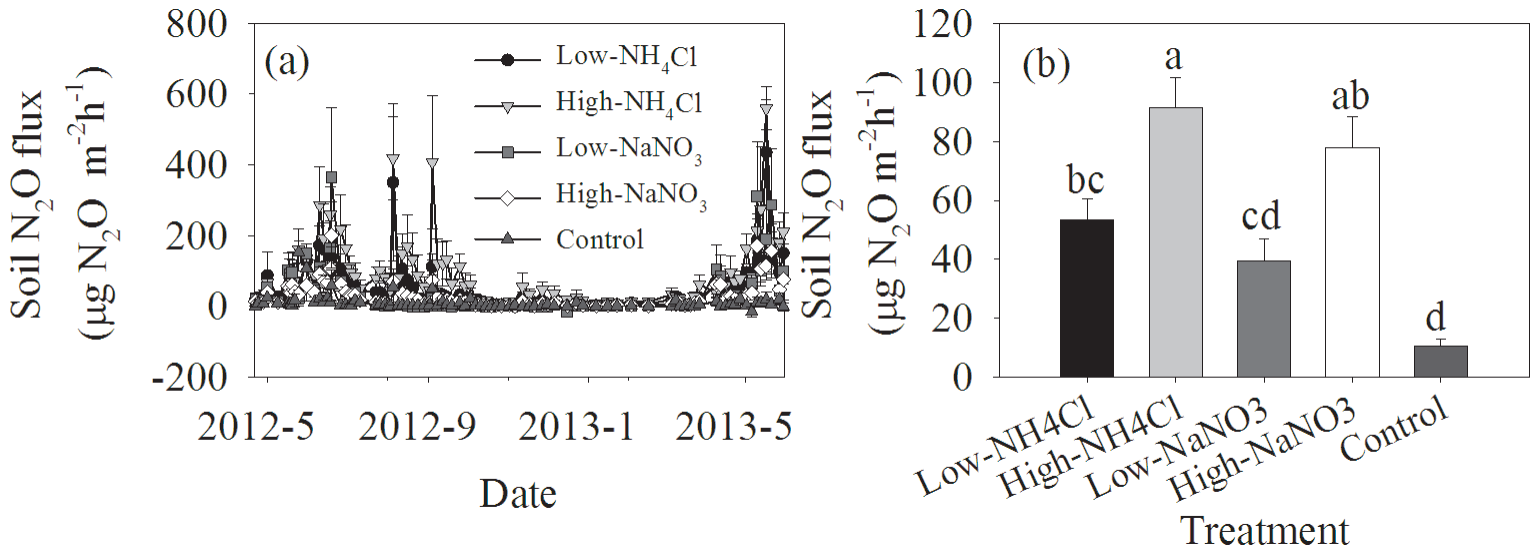
Temporal variations of soil N_2_O fluxes and their responses to N addition. Different letters above the columns mean significant differences between experimental treatments.

Soil N_2_O fluxes also showed a significant seasonality with the minimum occurring from early October to March next year (Fig. 4 a, Table 2, *P*<0.001). In the control, Soil N_2_O flux ranged from −15.26 to 559.30 µg N_2_O m^−2 ^h^−1^, with an average of 10.60 µg N_2_O m^−2 ^h^−1^ (Fig. 4b). Nitrogen addition produced obvious peaks of soil N_2_O emission, which was detected within one week after N addition (Fig. 4a). Soil N_2_O fluxes positively responded to N addition, and the promotion increased with the levels of N addition (Fig. 4b). In addition, there was a significant interaction between month and N treatment in the entire observation period (Table 2, *P*<0.001). For the same level of N addition, NH_4_Cl fertilizer had a higher promotion to soil N_2_O emission than NaNO_3_ fertilizer (Fig. 4b).

### Relationships between soil CH_4_ and N_2_O fluxes and soil properties

**Figure 5:**
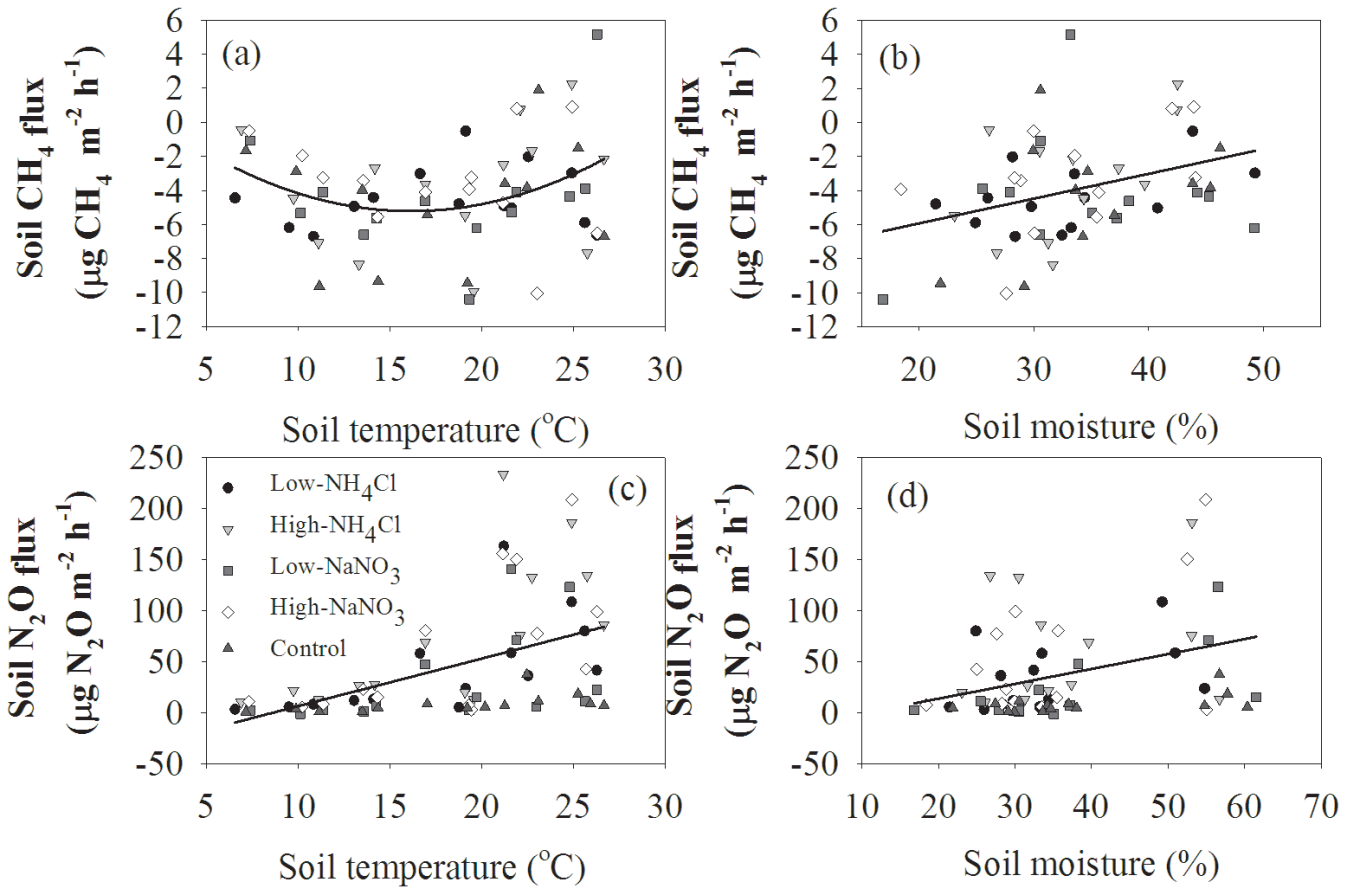
Relationships between soil CH_4_ and N_2_O fluxes, 5 cm soil temperature, and 10 cm soil moisture (n = 70).

Both soil CH_4_ and N_2_O fluxes were positively correlated with soil temperature at 5 cm depth and soil moisture at 10 cm depth (Fig. 5, Table 3). The relationships between soil CH_4_ fluxes and soil temperature and between soil CH_4_ fluxes and soil moisture could be well fitted with quadratic and linear equations, respectively (Fig. 5a–b, Table 3). Similarly, soil N_2_O fluxes were linearly correlated with soil temperature and soil moisture (Fig. 5c–d, Table 3).

**Table 3:** Regression models between soil CH_4_ and N_2_O fluxes and soil properties.

Flux	Soil variables^a^	Regression equation	R^2^	*P* value
CH_4_	T_s_	y = 0.028 T_S_^2^−0.90 T_S_+2.02	0.094	0.044
	M_S_	y = 0.15 M_S_−8.84	0.123	0.006
	NO_3_^−^	y = −1.28 NO_3_^−^ −1.77	0.21	0.004
	Combined	y = 0.17 M_S_−10.66	0.21	0.003
N_2_O	T_5_	y = 4.67 T_S_−40.11	0.25	<0.0001
	M_S_	y = 1.99 M_S_−29.76	0.10	0.001
	NO_3_^−^	y = 12.29 NO_3_^−^+10.58	0.22	0.005
	NH_4_^+^	y = 81.52 ln (NH_4_^+^)−85.34	0.37	<0.0001
	TDN	y = 9.26 TDN−93.50	0.17	0.008
	Combined	y = 0.01 NH_4_^+^+0.013 NO_3_^−^−0.041	0.50	<0.0001

a : T_S_ is soil temperature at 5 cm depth, M_S_ is soil moisture at 10 cm depth, NH_4_^+^, NO_3_^−^, and TDN are the concentrations of soil NH_4_^+^, NO_3_^−^, and TDN at 20 cm depth.

**Figure 6:**
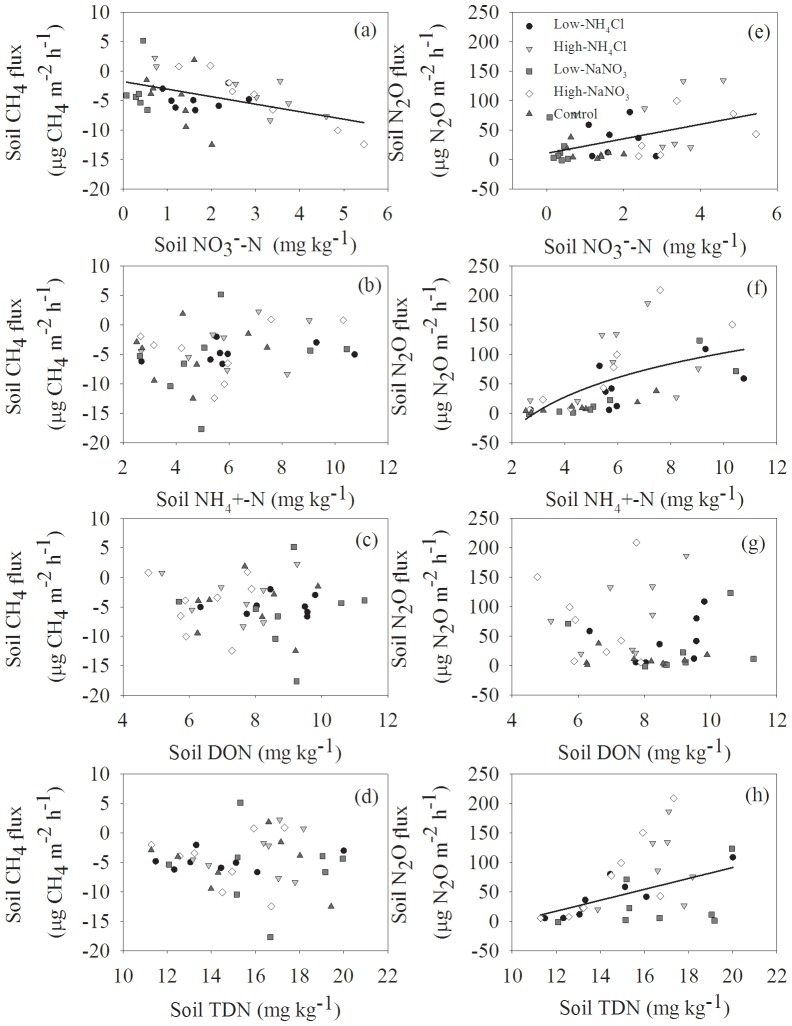
Relationships between soil CH_4_ and N_2_O fluxes and soil dissolved N concentrations (n = 40).

Soil CH_4_ fluxes were positively correlated with soil NO_3_^−^-N concentrations, whereas no significant correlations between soil CH_4_ fluxes and other dissolved N species were found (Fig. 6a–d, Table 3). Soil N_2_O fluxes were linearly correlated with soil NO_3_^−^-N and TDN concentrations (Fig. 6e, Fig. 6h, Table 3), and the relationship between soil N_2_O fluxes and soil NH_4_^+^-N concentrations was well fitted with a logarithm equation (Fig. 6f, Table 3).

## Discussion

### Effects of N addition on soil CH_4_ fluxes

The subtropical plantation soils can act as a sink of atmospheric CH_4_. The mean annual soil CH_4_ uptake in the control (0.49 kg CH_4_ ha^−1^ yr^−1^) was lower than those of subtropical plantation in Pingxiang (3.84 kg CH_4_ ha^−1^ yr^−1^) and Dinghushan of southern China (1.34 kg CH_4_ ha^−1^ yr^−1^) [5], [6] as well as that of subtropical rainforest in Australia (3.13 kg CH_4_ ha^−1^ yr^−1^) [3]. Except low level of NaNO_3_ treatment, the other three treatments decreased, on average, the rates of soil CH_4_ uptake by 18.38% to 41.04% relative to control (Fig. 3). The decrease in soil CH_4_ uptake caused by N addition in our site was higher than those of plantations in Dinghushan [5] and Heshan stations of southern China [8], despite the levels of N addition are similar in the three forest sites (120 vs. 150 kg ha^−1^ yr^−1^). This result indicated that the response of soil CH_4_ uptake to N addition was more sensitive in the northern subtropical plantations than in the southern subtropical plantations. This could be attributed to the lower soil N availability, lower atmospheric deposition rate, and the shorter duration of N application in QYZ, compared with the southern subtropical plantations [5], [8]. Furthermore, the subsurface mineral soils generally have higher capacity of oxidizing CH_4_ than surface litter layer [22], [24]. In our site, exogenous N input would directly affect the soil methanotrophic community as well as the amount of CH_4_ oxidation due to the lacking of litter layer.

Generally, atmospheric N deposition increases NH_4_^+^ accumulation and thereby inhibits CH_4_ uptake in the well-drained forest soils [8], [12], [43], despite contrasting results such as promotion and no effect have also been documented [44], [45]. In this study, we found that various levels and forms of N addition did not significantly change soil CH_4_ uptake over one year (Fig. 3). This could be related to the following third aspects. First, the short-term N fertilizers application did not significantly lead to soil NH_4_^+^-N accumulation (Fig. 2b), and no significant relationship between soil CH_4_ fluxes and soil NH_4_^+^-N concentrations was found (Fig. 6b). Whalen and Reeburgh [46] also concluded that N inputs did not influence CH_4_ uptake until they significantly increased soil NH_4_^+^ availability in the boreal forest soils. Although an inhibitory trend of soil CH_4_ uptake under the NH_4_^+^-N addition treatments was found, the competition and toxic inhibition of accumulated NH_4_^+^ did not occur over the short term. Second, N addition enhances the availability of NH_4_^+^ to soil nitrifiers, which will accordingly decrease the extent to which CH_4_ consumers are exposed to NH_4_^+^ [20]. A slight accumulation in soil NO_3_^−^-N concentration under NH_4_^+^-N fertilizer treatments indirectly supported our deduction (Fig. 3a). Third, we also found that soil NO_3_^−^-N accumulation could significantly promote soil CH_4_ uptake (Fig. 6a), which had been documented in the subtropical plantations of southern China [6]. Especially, the low level of NaNO_3_ treatment tented to reduce soil NO_3_^−^-N concentration, and thereby it slightly stimulated soil CH_4_ uptake (Fig. 2b, Fig. 3b). Moreover, stronger relationships were found between soil CH_4_ fluxes and soil NO_3_^−^-N concentrations than between soil CH_4_ fluxes and other soil dissolved N concentrations (Fig. 6), suggesting that soil NO_3_^−^ played a more important role in soil CH_4_ uptake than other soil dissolved N species in the subtropical plantation.

Soil CH_4_ flux is controlled by methanogens operating at anaerobic conditions and methanotrophs taking oxygen as a terminal electron acceptor [47]. Activities and population sizes of these microbial communities depend on a series of soil factors, including soil temperature, moisture, pH, substrate availability, and aeration of soil profile [19], [48], [49]. Soil CH_4_ uptake is dominated by an optimal soil temperature [50]. In our study, the optimal soil temperature was about 15°C (Table 3), and the capacity of soil methanotrophs to oxidize CH_4_ would decline when soil temperature was lower or higher than the threshold [51]. Also, soil moisture controls the mass flow of air and diffusion of atmospheric CH_4_ into the soil by altering the water filled pore space (WFPS) of soils [52]. We also found that soil CH_4_ fluxes under the N addition and control treatments were significantly related to soil moisture (Table 3). Based on the result of stepwise regression analysis, we found that the variation in soil CH_4_ uptake was less affected by soil moisture (Table 3). Because N addition did not change soil moisture (Fig. 1), we reasonably deduced that the variation of CH_4_ uptake elicited by N treatments was mainly attributed to the change in soil N availability.

### Effects of N addition on soil N_2_O fluxes

Our result showed that the subtropical slash pine plantation in QYZ exhibited a source of atmospheric N_2_O under natural conditions. The average soil N_2_O flux in the control (0.93 kg N_2_O ha^−1^ yr^−1^) was comparable with that of Heshengqiao station in Hubei province (0.71 kg N_2_O ha^−1^ yr^−1^) [53], but lower than that of Dinghushan station in South China (2.11 kg N_2_O ha^−1^ yr^−1^) [33]. In our study, NH_4_Cl and NaNO_3_ addition at rates of 40 and 120 kg N ha^−1^ yr^−1^ increased soil N_2_O emission by 403% to 762%. On the contrary, in the pine, mixed and evergreen broadleaved forests of Dinghushan station, NH_4_NO_3_ addition at rates of 50, 100 and 150 kg N ha^−1^ yr^−1^ only increased soil N_2_O fluxes by 38% to 58% [33]. These results indicated that the subtropical plantation had high turnover rates of soil N and sensitively responded to increased N deposition. The potential reasons include that the optimal hydrothermal conditions [54], low soil pH [55], and high clay content [39], which favor both soil nitrification and denitrification as well as soil N_2_O emission.

Except soil DON concentration, soil N_2_O fluxes were significantly correlated with concentrations of soil NH_4_^+^, NO_3_^−^, and TDN (Fig. 6), suggesting soil N_2_O flux was dominated by both soil nitrification and denitrification processes. Furthermore, the promotion of NaNO_3_ addition to N_2_O emission was slightly lower than that of NH_4_Cl addition (Fig. 4). Two potential mechanisms can be responsible for this phenomenon: (1) the high rates of NO_3_^−^ immobilization and nitrification [38], and the low denitrification potential are found in the same type of subtropical plantations [56]; and (2) temperature regulates soil N_2_O flux through influencing soil N_2_O-producing microorganisms, such as nitrifers and denitrfiers [57]. Soil moisture effects on soil N_2_O fluxes are a result of the limitation of O_2_ diffusion into the soil and the expansion of soil anaerobic microbial community [58]. The relatively high temperature in wet season was benefit for soil nitrifers and denitrfiers activities, which partly explained the seasonal variation of soil N_2_O fluxes with maximum occurring in between May and June (Fig. 4a). Because N addition did not change soil temperature and soil moisture (Fig. 1), the changes in soil N_2_O emission under N addition treatments were unlikely to be caused by the changes in soil temperature and soil moisture. Therefore, soil NH_4_^+^-N and NO_3_^−^-N concentrations were the dominant factors controlling soil N_2_O emission in our study, and could explain 49.9% of the temporal variability of soil N_2_O fluxes (Table 3).

## Conclusions

This study emphasizes the contrasting effects of oxidized NO_3_^−^ and reduced NH_4_^+^ inputs on the fluxes of CH_4_ uptake and N_2_O emission from a subtropical plantation soil based on high frequency observations. We found that N addition tended to inhibit soil CH_4_ uptake, and dramatically promoted soil N_2_O emission. Compared with NO_3_^−^-N fertilizer application, NH_4_^+^-N fertilizer application had a stronger inhibition to soil CH_4_ uptake and a stronger promotion to soil N_2_O emission. Also, both soil CH_4_ and N_2_O fluxes were driven by soil moisture and temperature, but soil inorganic N availability was a key integrator of soil CH_4_ uptake and N_2_O emission. Overall, short-term N addition has already changed soil CH_4_ and N_2_O fluxes, which indicated that the subtropical plantation soil was sensitive to N deposition input. In the future, the long-term observation of soil fluxes and the measurement of key microbial functional groups are necessary to clarify the mechanisms responsible for the coupling between soil CH4 and N2O fluxes.
